# Lessons to be popular: the chemical basis of aggregation in *Trypanosoma cruzi*-infected and non-infected Chagasic bugs

**DOI:** 10.1098/rsos.231271

**Published:** 2024-02-14

**Authors:** David Alavez-Rosas, Ana E. Gutiérrez-Cabrera, Leopoldo Cruz-López, Alex Córdoba-Aguilar

**Affiliations:** ^1^ Instituto de Ecología, Universidad Nacional Autónoma de México, Circuito exterior s/n, Ciudad Universitaria, Ciudad de México 04510, Mexico; ^2^ CONAHCYT- Instituto Nacional de Salud Pública Consejo Nacional de Ciencia y Tecnología, Avenida Universidad 655, Santa María Ahuacatitlán, 62100 Cuernavaca, Morelos, Mexico; ^3^ El Colegio de la Frontera Sur, Unidad Tapachula, Carretera Antiguo Aeropuerto Km. 2.5, Centro, 30700 Tapachula, Chiapas, Mexico

**Keywords:** Chagas disease, vectors, aggregation behaviour, semiochemicals, triatomines, parasitism

## Abstract

Aggregation is one of the most remarkable behaviours in the animal kingdom—a process that is usually governed by pheromones. Triatomines are blood-sucking bugs that act as vectors of *Trypanosoma cruzi*, the etiological agent of Chagas disease in mammals, including humans. Triatomines usually gather in roosting refuges by using aggregation pheromones of unknown chemical structure. In terms of vector control, one option to reduce triatomine–human contact is via capturing the insects into traps baited with lures based on such aggregation pheromones. As a first step towards this aim, we elucidated the aggregation pheromone in the triatomine *Triatoma pallidipennis*, using *T. cruzi*-infected and non-infected bugs. We used different extraction techniques and gas chromatography coupled to mass spectrometry for the identification. Also, two different bioassays were implemented for evaluating the attractant and arrestant activity of the pheromone. We found that *T. pallidipennis* produced short-chain aldehydes as attractants, and nitrogen-derived compounds as arrestants. We detected differences in the production and perception of these compounds according to whether animals were infected or not. These findings show that *T. cruzi* may influence triatomine chemical ecology and are promising tools for triatomine control.

## 1. Introduction

Aggregation pheromones have been identified in various gregarious insects, e.g. cockroaches [1], termites [2], stink bugs [3], bed bugs [4], bark beetles [5,6] and longhorn beetles [7,8]. This wide spanning occurrence has a fundamental reason: pheromones are key traits involved during communication in a number of processes such as mate choice, group foraging and collective gathering near the pheromone source (either by attracting conspecifics from a distance (e.g. attractant activity) or by inducing passing conspecifics to remain at the pheromone source (e.g. arrestant activity) [2,4]). These pheromones usually comprise a unique compound or a mix of compounds. While for the former, the compound works as an attractant and arrestant, for the latter, compounds are differentially used as attractants and arrestants [2,4].

In terms of public health, controlling blood-sucking insects has lagged behind and humanity is still at risk for a number of zoonotic diseases, Chagas disease being one example. Chagas disease is caused by *Trypanosoma cruzi*, which is vectored by triatomine bugs [9]. These bugs are gregarious and nocturnal insects that feed mostly on bird and mammal blood [10]. During daylight hours, these insects use different physical and chemical cues to orient towards refuges [11,12]. As for the chemical cues, triatomines are attracted to conspecific and heterospecific faeces [13–15], cuticular lipids [16,17] and footprints [18–20]. Moreover, despite these indications of an aggregation signal in these animals [14,15,21,22], there is no detailed information regarding the chemistry of an aggregation pheromone. Elucidating such chemical aspects can be key for behavioural-based triatomine control programmes [23]. Our background information for aggregation pheromones in triatomines is fragmented. First, short-chain aldehydes (C_6_–C_10_) have been found in faeces or in volatiles of aggregated insects which work as attractants [12,21,24]. Second, alkanes (C_18_–C_25_) have been found in faeces of *T. dimidiata* and *T. infestans* [12]. Third, two quinazolines were detected in faeces of *T. infestans*, which served to attract nymphs [22,25]. In terms of attraction, faecal compounds (3-methylbutyric acid, hexanoic acid, 2,3-butanediol, acetic acid and acetamide) attracted *T. infestans*, *Panstrongylus megistus* and *T. brasiliensis* [10]. Finally, lipids (hexadecanoic acid, octadecanoic acid and octacosanoic acid) and fatty alcohols (eicosanol and docosanol) from the bug cuticle, mediated aggregation in *T. infestans* [16,17]. It remains unexplored whether all these compounds work as an aggregation pheromone.

One overlooked aspect in the ecological study of insect vectors is the possibility that their vectored pathogens may affect them to increase transmission to final hosts [26–28]. Some data suggest that this may be the case for the case of triatomines and *T. cruzi* in terms of their chemical ecology. For example, the aggregation and geotaxis of males and females of *T. infestans*, have been analysed, and authors concluded that infected bugs reinforced their gregariousness [29]. Additionally, it has been observed significant differences between sensilla patterns of infected and non-infected insects have been observed [30]. More direct evidence of possible parasite-driven effects indicated that infected bugs were more active and likely to detect human odour than non-infected bugs [31]. To what extent the parasite influences the production and perception of aggregation pheromone in triatomines remains unclear.

In this paper, we used *T. pallidipennis* triatomine bugs as a study subject. This is not circumstantial: *T. pallidipennis* is responsible for *ca* 74% of Chagas vectorial transmission in Mexico. This is because it has a wide distribution, high abundance compared with other triatomines, close contact with humans and high *T. cruzi* infection rates [32]. We set the following aims: (i) to unravel the possible origin of the pheromone components as well as the component associated with the arrestant behaviour; (ii) to assess a mixture of alternative compounds that may act as an aggregation pheromone; and (iii) to investigate whether a *T. cruzi* infection leads to a change in the production and perception of the pheromone.

## 2. Results

### 2.1. Bioassays with faeces, aggregated insects and extracts

#### 2.1.1. Y-tube bioassays

**Figure 1.**
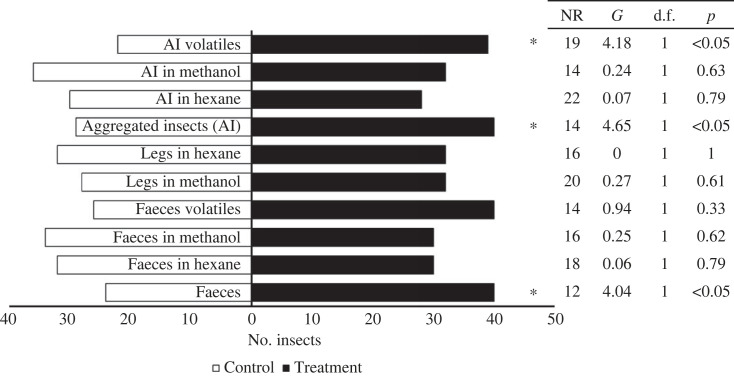
Responses of individual fifth-instar nymphs of *T. pallidipennis* in a Y-tube bioassay. Eighty repetitions per treatment were performed.

Fifth-instar nymphs of *T. pallidipennis* were attracted to volatiles emitted by faeces (*G* = 4.04, d.f. = 1, *p* < 0.05), aggregated insects (*G* = 4.64, d.f. = 1, *p* < 0.05), and headspace volatiles extracted from insects (*G* = 4.18, d.f. = 1, *p* < 0.05, figure 1). Similar results were observed with adults, fourth- and third-instar nymphs (electronic supplementary material, figures SF1, SF2 and SF3). Neither infected adults nor fifth-instar nymphs were attracted to any treatment (electronic supplementary material, figures SF4 and SF5).

#### 2.1.2. Rectangular cage bioassays

**Figure 2.**
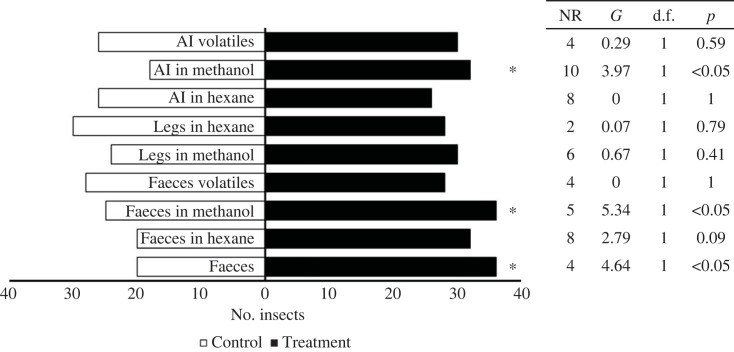
Responses of individual fifth-instar nymphs of *T. pallidipennis* in a rectangular cage bioassay. Sixty repetitions per treatment were performed.

**Figure 3.**
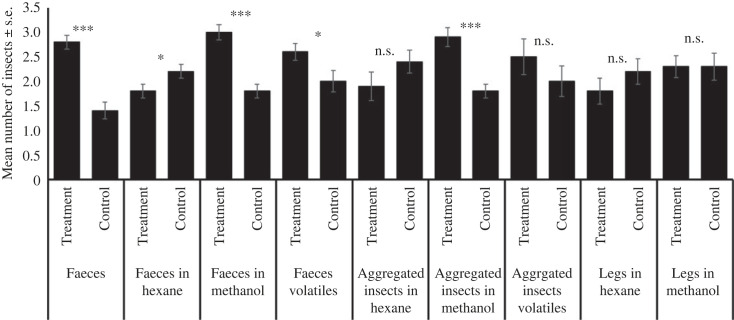
Responses of five fifth-instar nymphs of *T. pallidipennis* in a rectangular cage bioassay. Ten repetitions per treatment were performed. Faeces (*t* = −6.64, d.f. = 18, *p* < 0.001), faeces in hexane (*t* = 2.12, d.f. = 18, *p* < 0.05), faeces in methanol (*t* = −6.00, d.f. = 18, *p* < 0.001), faeces volatiles (*t* = −2.25, d.f. = 18, *p* < 0.05), aggregated insects in hexane (*t* = 1.41, d.f. = 18, *p* = 0.18), aggregated insects in methanol (*t* = −4.92, d.f. = 18, *p* < 0.001), aggregated insects volatiles (*t* = −1.10, d.f. = 18, *p* = 0.28), legs in hexane (*t* = 1.13, d.f. = 18, *p* = 0.27), legs in methanol (*t* = 14.03, d.f. = 19, *p* = 0.81).

Individual fifth-instar nymphs moved towards artificial shelters marked with faeces (*G* = 4.64, d.f. = 1, *p* < 0.05), extracts of faeces in methanol (*G* = 5.34, d.f. = 1, *p* < 0.05) and extracts with methanol from aggregated insects (*G* = 3.97, d.f. = 1, *p* < 0.05). However, other treatments were not attractive to the bugs (figure 2), which applied to adults, fourth- and third-instar nymphs (electronic supplementary material, figures SF6, SF7 and SF8). Additionally, fifth-instar infected nymphs were attracted to their faeces (*G* = 3.99, d.f. = 1, *p* < 0.05; electronic supplementary material, figure SF9), infected adults were attracted to their faeces (*G* = 4.14, d.f. = 1, *p* < 0.05), to the methanol extracts of their faeces (*G* = 4.30, d.f. = 1, *p* < 0.05) and aggregated insects (*G* = 4.14, d.f. = 1, *p* < 0.05; electronic supplementary material, figure SF10). Regarding bioassays with a group of bugs, the responses of bugs to faeces was similar throughout the day and among nymphs and adults (electronic supplementary material, figures SF11 and SF12). Fifth-instar nymphs were attracted to faeces (*t* = −6.64, d.f. = 18, *p* < 0.001), faeces in hexane (*t* = 2.12, d.f. = 18, *p* < 0.05), faeces in methanol (*t* = −6.00, d.f. = 18, *p* < 0.001), faeces volatiles (*t* = −2.25, d.f. = 18, *p* < 0.05), aggregated insects in methanol (*t* = −4.92, d.f. = 18, *p* < 0.001, figure 3). Infected nymphs were attracted to faeces (*t* = −2.49, d.f. = 18, *p* < 0.05), faeces in methanol (*t* = 3.35, d.f. = 18, *p* < 0.01), aggregated insects in methanol (*t* = −2.79, d.f. = 18, *p* < 0.05) and to aggregated insects volatiles (*t* = −2.71, d.f. = 18, *p* < 0.05; electronic supplementary material, figure SF13). Similar results were found with both infected and non-infected adults (electronic supplementary material, figures SF14 and SF15).

### 2.2. Chemical analysis

Eleven volatile compounds were found in faeces and aggregated insects of non-infected bugs (table 1). The most abundant compounds were 2-ethyl-1-hexanol (29.91%) for the faeces and octanal (31.78%) for aggregated insects. Other aldehydes, heterocyclic compounds and carboxylic acids were found in lower proportions. The release rate of compounds was found in the order of micrograms per week, a total of 20.33 µg week^−1^ and 10.28 µg week^−1^ of volatiles from faeces and aggregated insects, respectively, were captured (electronic supplementary material, table ST1). Twelve volatile compounds were found in faeces and aggregated insects of infected bugs, most abundant compounds were 2-ethyl-1-hexanol (20.98%) and nonanal (22.54%) for faeces and the same compounds (20.96% and 24.68%, respectively) for aggregated insects (table 1). A total amount of 22.95 µg week^−1^ and 44.73 µg week^−1^ of volatiles were released from faeces and aggregated insects of infected bugs. Similar results were found in the solid-phase microextraction (SPME) fibres; however, we found pentanal, hexanal and heptanal in the faeces (electronic supplementary material, table ST2). SPME volatiles from infected insects were similar to those from non-infected insects.

**Table 1.** Percentages (% ± s.e.) of volatiles from faeces (collected from 150 fifth-instar nymphs in one month) and aggregated insects (50 fifth-instar nymphs) of *T. pallidipennis*. RT, retention time; KRI, Kovats retention index; LRI, library retention index.

entry	RT	KRI	LRI	name	faeces		aggregated insects	
					non-infected	infected	non-infected	infected
1	6.41	1006	1005	octanal^a,b^	14.04 ± 4.79	3.47 ± 0.39	31.78 ± 7.50	2.77 ± 0.36
2	6.66	1029	1028	2-ethyl-1-hexanol^a,b^	29.91 ± 5.84	20.98 ± 1.97	19.85 ± 7.48	20.96 ± 1.17
3	7.48	1100	1100	*n*-undecane^a,b^		6.80 ± 0.62		6.92 ± 0.91
4	7.50	1102	1102	nonanal^a,b^	9.43 ± 1.20	22.54 ± 2.25	12.25 ± 2.01	24.68 ± 2.01
5	8.17	1165	1164	(*E*)−2-nonenal^a^		4.29 ± 0.87		3.47 ± 0.73
6	8.53	1200	1200	*n*-dodecane^a,b^		4.83 ± 0.82		4.80 ± 0.59
7	8.66	1211	1210	decanal^a,b^	7.55 ± 1.51	11.43 ± 1.05	7.50 ± 0.86	12.47 ± 1.35
8	9.04	1249	1248	benzothiazole^a^		2.66 ± 0.57		2.92 ± 0.32
9	9.10	1256	1255	nonanoic acid^a,b^	5.41 ± 1.32		4.93 ± 0.81	
10	9.25	1271	1270	caprolactam^a,b^	4.28 ± 1.32		7.34 ± 1.44	
11	9.60	1300	1300	*n*-tridecane^a,b^		7.58 ± 0.86		7.04 ± 0.59
12	10.11	1361	1360	decanoic acid^a,b^	10.46 ± 1.29	3.36 ± 0.76	7.75 ± 1.35	2.46 ± 0.33
13	10.15	1360	1359	2-methylquinazoline^a^	5.66 ± 1.04		1.69 ± 0.77	
14	10.59	1400	1400	*n*-tetradecane^a,b^		6.30 ± 1.12		4.71 ± 0.53
15	10.57	1411	1410	2,4-dimethylquinazoline^a^	3.45 ± 0.56		1.23 ± 0.49	
16	11.12	1473	1472	1-dodecanol^a,b^	6.11 ± 1.78	5.76 ± 1.13	4.03 ± 1.95	6.79 ± 0.89
17	11.84	1560	1559	dodecanoic acid^a^	3.70 ± 1.74		1.65 ± 0.63	

^a^Identification based on comparison of spectral and retention database of NIST library (https://webbook.nist.gov/chemistry/name-ser/).

^b^Identification based on comparison with the standard.

Volatiles from faeces and from aggregated insects were mostly the same, yet qualitative differences were found between infected and non-infected insects: *n*-undecane, *n*-dodecane, *n*-tridecane, *n*-tetradecane, dodecanol, benzothiazole and 2-(*E*)-nonenal were found only in infected insects. Meanwhile, nonanoic acid, caprolactam, 2-methylquinazoline, 2,4-dimethylquinazoline and dodecanoic acid were only found in non-infected insects.

Hexane extracts of faeces showed the presence of cholesterol (94%) and lanostherol (6%), on its part, extracts of aggregated insects possessed the same compounds in 91.5% and 8.5%, respectively. Meanwhile, extracts of legs showed 11 compounds with cholesterol as the most abundant compound (55.2%), and hydrocarbons with lower proportions (table 2). The amounts found in insects (in ng per insect) are shown in electronic supplementary material, table ST3. Extracts of infected insects presented qualitative differences to those of non-infected. The histamine derivative, 9-nonadecenal and three non-identified compounds were absent in extracts from infected insects (electronic supplementary material, table S4).

**Table 2.** Relative amount (% ± s.e.) of compounds of *T. pallidipennis* from extracts of faeces (collected from 150 fifth-instar nymphs in one month), legs (five insects) and aggregated insects (50 fifth-instar nymphs). RT, retention time; KRI, Kovats retention index; LRI, library retention index.

entry	RT	KRI	LRI	compound	faeces in hexane	faeces in methanol	legs in methanol	legs in hexane	AI in hexane	AI in methanol
1	8.88	1282	1281	valine^a,b^			10.19 ± 2.27			
2	10.525	1450	1449	histamine^a,b^		4.22 ± 2.83				
3	11.01	1502	1502	proline^a,b^			25.53 ± 1.34			
4	13.21	1769	1768	histidine^a,b^		22.16 ± 6.71				16.81 ± 6.09
5	14.035	1879	1878	histamine derivate		2.48 ± 1.63				7.66 ± 4.29
6	14.395	1929	1928	hexadecanoic acid methyl ester^a^			5.52 ± 0.33			
7	14.69	1971	1970	hexadecanoic acid^a^		4.71 ± 0.80	0.41 ± 0.18			
8	15.57	2100	2100	linoelaidic acid^a^			5.00 ± 0.20			
9	15.605	2106	2105	*cis*-10-octadecenoic acid methyl ester^a^			13.51 ± 0.56			
10	15.74	2127	2126	octadecanoic acid methyl ester^a^			4.16 ± 0.50			
11	15.875	2148	2147	9-nonadecenal^a^		8.11 ± 1.96				
12	15.895	2151	2150	γ-palmitolactone^a^			4.11 ± 2.50			
13	16.02	2170	2170	n-octadecanoic acid^a^		3.76 ± 0.78				
14	16.63	2268	2267	9-eicosenal^a^			0.88 ± 0.07			
15	16.75	2287	2287	2-eicosanone^a^			0.32 ± 0.02			
16	16.83	2300	2300	*n*-tricosane^a,b^				0.85 ± 0.19		
17	17.015	2331	2331	heneicosanal^a^			2.90 ± 0.22			
18	17.165	2357	2356	not identified			0.45 ± 0.09			
19	17.325	2383	2383	not identified			1.11 ± 0.26			
20	17.82	2464	2463	not identified			2.55 ± 0.13			
21	17.885	2474	2474	not identified			0.16 ± 0.01			
22	17.98	2495	2495	not identified			0.56 ± 0.01			
23	18	2500	2500	*n*-pentacosane^a,b^				1.58 ± 0.36		
24	19.34	2672	2671	not identified			2.52 ± 0.43			
25	19.39	2678	2677	9-heptacosene^a^				3.04 ± 1.39		
26	19.44	2684	2683	1-heptacosene^a^				0.91 ± 0.39		
27	19.575	2700	2700	*n*-heptacosane^a,b^				2.54 ± 0.29		
28	20.76	2818	2818	not identified				1.09 ± 0.09		
29	21.725	2900	2900	*n*-nonacosane ^a,b^				6.28 ± 0.79		
30	23.175	3000	3000	*n*-triacontane ^a,b^				1.17 ± 0.29		
31	24.055	3051	3050	octacosanal ^a^				2.46 ± 0.89		
32	24.94	3103	3103	*n*-hentriacontane^a,b^				24.8 ± 2.39		
33	26.025	3173	3172	cholesterol^a,b^	94.03 ± 0.09	48.69 ± 9.36	20.11 ± 3.11	55.2 ± 3.89	91.54 ± 1.15	69.54 ± 8.75
34	26.26	3188	3187	lanostherol^a^	5.97 ± 0.09	5.86 ± 1.17			8.46 ± 1.12	5.99 ± 0.67

^a^Identification based on comparison of spectral and retention database of NIST library (https://webbook.nist.gov/chemistry/name-ser/).

^b^Identification based on comparison with the standard.

Methanol extracts of faeces and aggregated insects had cholesterol as the most abundant compound; however, they showed the presence of histamine, histidine and histamine-related compound and carboxylic acids in less proportion. Extracts of infected insects showed similar proportions and amounts of compounds (electronic supplementary material, tables ST4 and ST5).

### 2.3. Bioassays with standards and blends

#### 2.3.1. Y tube

**Figure 4.**
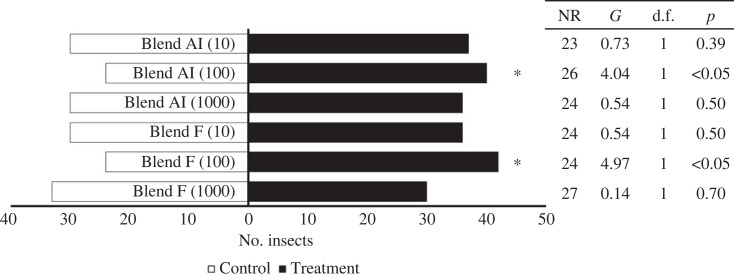
Responses of fifth-instar nymphs of *T. pallidipennis* in a Y-tube bioassay using blends of compounds from the faeces (blend F) and aggregated insects (blend AI) at three different concentrations. Ten repetitions per treatment were performed.

Both blends were attractive for fifth-instar nymphs at 100 ng ml^−1^, blend F (*G* = 4.97, d.f. = 1, *p* < 0.05), blend AI (*G* = 4.04, d.f. = 1, *p* < 0.05; figure 4). Only the blend corresponding to faeces proportions (blend F) was attractive for adults (*G* = 5.97, d.f. = 1, *p* < 0.05; electronic supplementary material, figure SF16). Standards used in the F blend (faeces blend) were: hexanal (10%), heptanal (10%), octanal (10%), 2-ethyl-1-hexanol (20%), nonanal (10%), decanal (10%), nonanoic acid (5%), caprolactam (5%), decanoic acid (10%) and 1-dodecanol (10%). Meanwhile, for the blend AI (aggregated insects) proportions were: hexanal (5%), heptanal (5%), octanal (5%), 2-ethyl-1-hexanol (20%), nonanal (20%), decanal (15%), nonanoic acid (5%), caprolactam (5%), decanoic acid (10%) and 1-dodecanol (10%). When standards of compounds were evaluated individually at 100 ng µl^−1^, fifth-instar nymphs preferred the control (hexane) over caprolactam (*G* = 10.47, d.f. = 1, *p* < 0.01) and decanoic acid (*G* = 3.97, d.f. = 1, *p* < 0.05). Only nonanal was attractive to nymphs (*G* = 4.17, d.f. = 1, *p* < 0.05; electronic supplementary material, figure SF17). Adults were attracted to octanal (*G* = 6.11, d.f. = 1, *p* < 0.05; electronic supplementary material, figure SF18).

#### 2.3.2. Rectangular cage

Single bugs were arrested by the combination of histidine + histamine (*G* = 4.30, d.f. = 1, *p* < 0.01), and histidine + histamine + cholesterol (*G* = 4.64, d.f. = 1, *p* < 0.01); responses to other binary combinations or to single compounds were not statistically significant to the control (electronic supplementary material, figure SF19). Similar results were found with adults (electronic supplementary material, figure SF20).

**Figure 5.**
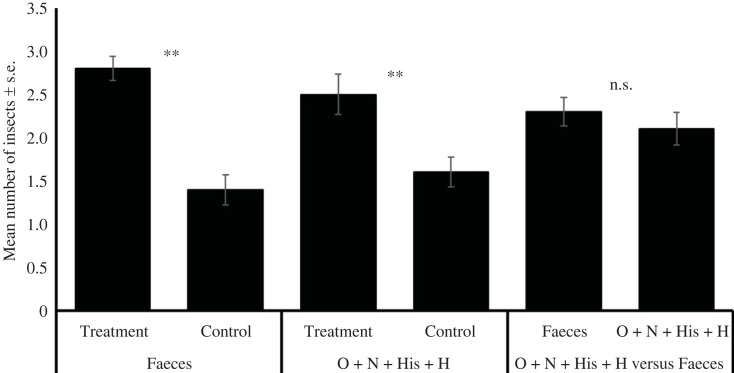
Responses of five fifth-instar nymphs of *T. pallidipennis* in the rectangular cage bioassay. Ten repetitions per treatment were performed. Faeces (*t* = −3.25, d.f. = 18, *p* < 0.01), O + N + His + H (*t* = −6.64, d.f. = 18, *p* < 0.01), faeces versus O + N + His + H (*t* = −0.84, d.f. = 18, *p* = 0.41).

In the experiments using a group of five insects, the combination of histidine + histamine + nonanal + octanal was attractive to non-infected (*t* = −6.64, d.f. = 18, *p* < 0.01) and infected fifth-instar nymphs (*t* = −3.25, d.f. = 18, *p* < 0.01); and bugs did not prefer faeces over the four-component blend (figure 5 and electronic supplementary material, figure SF21). Similar results were found with non-infected and infected adults (electronic supplementary material, figures SF22 and SF23).

### 2.4. Semi-field bioassays

**Figure 6.**
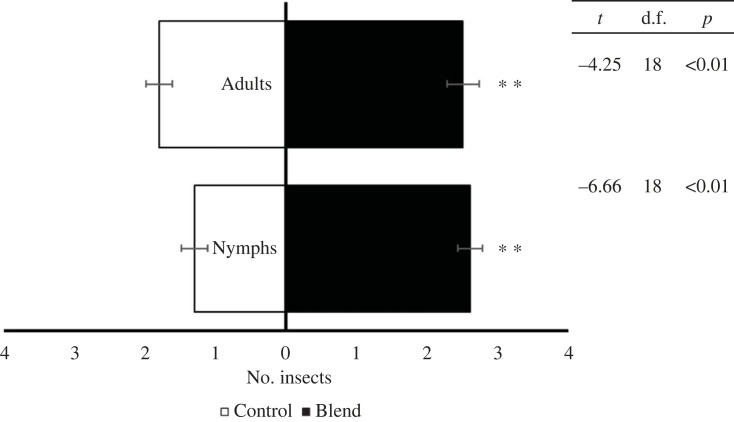
Responses of a group of five non-infected insects in a semi-field bioassay. Ten repetitions per treatment were performed. Adults of *T. pallidipennis* (*t* = −3.50, d.f. = 18, *p* < 0.01), nymphs of *T. pallidipennis* (*t* = −2.85, d.f. = 18, *p* < 0.01).

Non-infected adults were attracted to the four-component blend (octanal, nonanal, histidine and histamine) in semi-field experiments (*t* = −3.50, d.f. = 18, *p* < 0.01), the same result was observed for non-infected fifth-instar nymphs of *T. pallidipennis* (*t* = −2.85, d.f. = 18, *p* < 0.01) (figure 6).

## 3. Discussion

We demonstrated that in individual-bug bioassays, non-infected *T. pallidipennis* nymphs and adults were attracted to volatiles from faeces and aggregated conspecifics (yet infected bugs were not attracted). Moreover, better responses were observed in group-bioassays regardless of the infection status. This difference may be explained by the fact that triatomines take group-based rather than individual-based decisions. In this regard, the presence of conspecifics can guide individuals toward refuges, thereby reducing exposure to predators through improving refuge search efficiency [33]. As for the variation in chemical composition, it is possible that attractive volatile compounds from faeces are produced after a biochemical process because they were not found in the hexane or methanol extracts. This implies an *in situ* production probably microorganism-mediated or due to a chemical decomposition [34].

Arrestant compounds were present in methanol extracts from faeces and from places where insects were aggregated. Nevertheless, non-active compounds were also detected in hexane extracts of legs, faeces or aggregated insects. Non-infected and infected bugs, aggregated in shelters baited with methanol extracts from faeces. This observation echoes previous findings from *T. infestans* [22]. However, we noticed that these authors did not test the individual compounds found in the polar extracts. Historically, the investigation of the arrestant activity of the aggregation pheromone of triatomines has lagged behind, maybe because the attractive volatile component of the pheromone implies more field applications as a lure for traps. To our knowledge, this is the first work that aims to study both functional aspects (arrestant and attractant) of the pheromone for a triatomine.

Qualitative differences as well as the total amount of volatiles and solvent extracts could be associated with the *T. cruzi* infection, and thereby influence the gregariousness of infected insects [29]. Several questions remain unclear and deserve further attention. First, what is the link between parasite infection and volatile production? Since we used dry faeces to collect the volatiles, the parasite does not survive in such conditions, yet it may still participate in their decomposition. We did not test all the identified compounds, due to their reduced availability, thus, more studies are needed to know the effect of single compounds not evaluated here. Identified short-chain aldehydes have been previously reported as attractants of blood-sucking arthropods [23,24]. We decided to use static and dynamic collection techniques to make volatile collection more robust. Pentanal, hexanal and heptanal were only detected in the SPME experiments due to the solvent cut we used in the gas chromatography–mass spectrometry (GC-MS) analysis of solvent extracts. Besides only octanal and nonanal were attractive for *T. pallidipennis*, thus further work should be addressed to determine the influence of other short-chain aldehydes (e.g. 2-(E)-nonenal) in the aggregation behaviour of this insect. Second, does parasite's chemical influence affect its fitness? If the response to this question is yes, then such influence may induce other infected bugs to find the refuges to escape from predators. This is not clear, so it awaits further experiments to test it.

Histidine and histamine act as arrestants for *T. pallidipennis*. This is not new: the presence of these compounds as part of the aggregation pheromone for other blood-sucking insects has already been reported [4]. In the context of triatomines, we hypothesize that these compounds indicate the presence of a host to the insects, because amino acids and derivatives are part of the degradation of the ingested blood. Again, future tests should clarify this. Moreover, the blend of octanal, nonanal, histidine and histamine attracts and arrests *T. pallidipennis* irrespective of their developmental stage and infection status, in a similar fashion as the natural aggregation pheromone would produce [4]. However, we blended these compounds in a different ratio of the natural blend and it was attractive for the insects. This suggests that these compounds are not properly a signal (pheromone), but that serve as cues that communicate the presence of the kissing bugs (aldehydes) or host (amino acid derivatives). This idea is supported by recent findings of short-chain aldehydes found in faeces of other triatomines [12,19,22], and studies that demonstrate that faeces attract insects from the same as well as different species [13,15].

Finally, how do our findings translate into Chagas control? Our results can inform about means to prevent infected bugs from reaching mammal hosts: while octanal and nonanal act as attractants, histidine and histamine act as arrestants for *T. pallidipennis* adults and nymphs (infected and non-infected bugs). Moreover, we also found that different ratios of compound in the blend attract the bugs, which implies that compounds in faeces act as cues rather than signals.

## 4. Material and methods

### 4.1. Triatoma pallidipennis

We used third-, fourth- and fifth-stage nymphs and adults, maintained under controlled temperature, humidity and photoperiod (28 ± 2°C, 60 ± 5% RH and 12 : 12 h). Bugs were obtained from colonies from the Instituto Nacional de Salud Pública (INSP) located in Cuernavaca City, Mexico. This study was conducted between November 2020 and November 2022.

### 4.2. Trypanosoma cruzi

*Trypanosoma cruzi* were kept in specific facilities of the INSP. We used the *T. cruzi* strain ITRI/MX/12/MOR [35]. Parasites were maintained at 28°C in liver infusion tryptose (LIT) culture media. LIT medium was supplemented with fetal bovine serum 5% (SFB) and Hemin with a proportion 1 : 200 (LIT-SH). Parasites were used in the epimastigotes stage in exponential growth phase. A Neubauer chamber was used to determine the number of parasites per millilitre. To determine the number of parasites used it was necessary to multiply the number of parasites counted by 10^4^ due to the dilution factor. For feeding/infection of triatomines, we used 4 × 10^6^ parasites ml^−1^.

#### 4.2.1. Bug infection with *Trypanosoma cruzi*

Third-instar nymphs (unfed for 25–30 days after moulting) were infected using artificial feeders. For this, parasites were removed from the culture medium, and those parasites that were recovered were resuspended in blood used to feed triatomines. Before resuspension, it was necessary to separate the plasma from the haematocrit [36]. Then, plasma was collected from the surface and inactivated at 55°C [37]. Haematocrit was washed once with saline solution and centrifuged at 2500 r.p.m. for 10 min. Once the plasma was inactivated, the washed haematocrit was added. Finally, parasites were added. To confirm the infection, a faecal sample was obtained from each insect, a drop of isotonic saline solution was placed on a microscope slide, then the expulsion of faeces or urine was provoked by rectal stimulation and gentle abdominal compression of the insect. The sample was observed under a microscope at ×40 magnification. The whole sample was searched for the presence of parasites, considering insects positive for *T. cruzi* when they presented at least one parasite in the sample. The parasites were found in the form of blood trypomastigotes, epimastigotes and transitional (spheromastigotes).

#### 4.2.2. Faeces collection

Faeces from 100 fifth-instar nymphs were collected as previously described with slight modifications [12]. Bugs were placed in a plastic bottle with a filter paper at the bottom. Filter papers impregnated with dry faeces were maintained in 100 ml sealed glass vials at 30°C and 60 ± 5% relative humidity until used. Dry (10 days after excretion) faeces were used for bioassays. Volatiles from faeces were collected from filter papers 10 days post-excretion.

#### 4.2.3. Headspace collection of volatile from faeces and aggregated insects

##### 4.2.3.1. Dynamic aeration

Volatiles from faeces of insects were collected by placing 50–70 impregnated filter papers (10 cm diameter; Whatman International, Maidstone, UK) in a glass aeration chamber (1 l glass flask) with a charcoal-filtered airstream (1 l min^−1^). Headspace volatiles were collected in a glass volatile collection trap (8 mm internal diameter (ID) × 100 mm length) containing 100 mg of Tenax adsorbent (Merck KGaA, Darmstadt, Germany) during 60 days. An identical aeration chamber with clean filter papers was used as control. Volatiles were eluted every 24 h, using 500 µl of dichloromethane (high-performance liquid chromatography (HPLC) grade; J.T. Baker, Phillipsburg, NJ, USA). Headspace extracts were mixed to a final volume of 30 ml and volatile samples were slowly evaporated under a gentle stream of nitrogen to a final concentration of 50 µl and stored at −20°C until further use. We also collected volatiles from 150 aggregated insects in a 1 l aeration chamber, following the same methodology as for the faeces.

##### 4.2.3.2. Solid-phase microextraction experiments

We performed the sampling of the faeces and volatiles from aggregated insects using the solid-phase microextraction (SPME) technique. Faeces (1 g) or aggregated insects (50 fifth-instar nymphs) were placed separately in 100 ml glass containers (15 cm long × 6 cm diameter) and sealed with aluminium foil. An identical but empty glass container sealed with aluminium foil was used as the control. We sampled the volatiles with a polydimethylsiloxane/divinylbenzene-coated SPME fibre (film thickness 65 µm, Supelco, Toluca, Mexico). Sampling was performed by inserting the SPME needle through the aluminium foil into the glass container, and volatiles were allowed to be adsorbed onto the SPME fibre for 24 h. The SPME fibre was subsequently removed from the glass container, and the volatiles were desorbed inside the heated injection port of a gas chromatograph for 2 min. All volatile collections were performed in a room at 25 ± 2°C and 50–60% RH. Five replicates per treatment were performed. Before each collection, the SPME fibres were heated at 250°C for 15 min to clean any impurities.

Solvent extractions of bugs' hydrocarbons, faeces and places where bugs were aggregated were as follows.

##### 4.2.3.3. Bugs’ hydrocarbons

Bugs were frozen at −20°C before analysis. The hydrocarbons from the insects were prepared by washing the six legs of four fifth-instar nymphs in 3 ml of hexane or methanol (HPLC grade, Aldrich, Toluca, México) separately, concentrated to 50 µl using a gentle stream of dry N_2_ and stored at −20°C until analysis. As this extract was obtained from legs, chemical profile could be associated with footprints.

##### 4.2.3.4. Compounds from faeces

We extracted compounds from 100 mg of faeces in 1 ml of hexane or methanol (HPLC grade, Aldrich, Toluca, México) separately during 10 min. Then we filtered the solution, and the solvent was concentrated to 50 µl using a gentle stream of dry N_2_ and stored at −20°C until analysis.

##### 4.2.3.5. Compound from places where bugs were aggregated

Chemicals left by 50 fifth-instar nymphs, during one month, on a 1 l glass chamber were extracted by washing the feeders with two fractions of 5 ml each of n-hexane or methanol (HPLC grade, Aldrich, Toluca, México) separately. The extracts were concentrated to 50 µl using a gentle stream of dry N_2_ and stored in a freezer at −20°C until analysis.

#### 4.2.4. Chemical analysis

Compounds from extracts and SPME fibres were analysed using a gas chromatograph (Shimadzu GC-2010 plus) coupled to a triple–quadrupole mass spectrometer (Shimadzu TQ8040) using the electron-impact ionization (EI) at 70 eV, 250°C. A DB-5 fused silica capillary column (30 m × 0.25 mm ID) was temperature programmed from 50°C (held for 2 min) to 280°C at 15°C min^−1^; it was then held at 280°C for 10 min. The temperature of the injector was held at 250°C. Helium was used as the carrier gas at a constant flow of 1 ml min^−1^. Compounds were identified by comparison of retention indices (Kovats and linear), and mass spectral profiles using the National Institute of Standards and Technology (NIST) library. Identifications were confirmed by comparison of retention times and mass spectra using synthetic standards. Synthetic compounds (95% pure) were obtained from Sigma-Aldrich-Fluka (Toluca, Mexico). The relative amount (percentage) of a given component was calculated relative to the sum of all areas under the peaks. The amounts of compounds from insects and the release rates of compounds from faeces and from aggregated insects were quantified using the external standard method. We used the corresponding standard for the construction of the calibration curve to the quantitation of available compounds. When no standard was available, we used a chemically similar compound to the construction of the calibration curve.

#### 4.2.5. Laboratory bioassays

We evaluated two types of bioassays in the laboratory, in which we could evaluate the volatile part (Y-tube) and the non-volatile part (rectangular cage) of the aggregation pheromone. Solvents (HPLC grade) and available standards (greater than 99% purity) were obtained from Sigma Aldrich (Toluca, Mexico); all substances were used with no further purification.

##### 4.2.5.1. Y tube

The behavioural response of the bugs to the volatile part of different treatments was evaluated in the Y-tube olfactometer (Y-shaped glass tube 2.5 cm in diameter; the base and the two arms of the olfactometer were each 12 cm in length). Each arm was attached to a flow-meter and an odour source container, which consisted of a glass chamber (4.5 cm in diameter and 15 cm in height), and in the case of aggregated insects, one arm was attached to a 1 l glass chamber containing 150 fifth-instar nymphs. Activated charcoal-filtered air at a rate of 0.5 l min^−1^, regulated by flow-meters (Gilmont Instruments, Barnant Co., Barrington, IL, USA), was pushed into the chambers containing the treatments. The air was humidified by passing through a water flask before introduction into the olfactometer. A single bug was introduced into the central section of the Y-tube olfactometer and observed for 5 min. A choice was recorded when the bug chooses one of the arms and stayed there for at least 5 s. After 5 min, if the insect did not make a choice, it was considered as non-responder. The position of odour source in the sample chamber was changed after each trial to eliminate directional bias. We performed the bioassays between 8.00 and 20.00 in a room at 24–26°C and 50–60% RH. Illumination was provided by a red LED coloured light bulb (Phillips, 8 W, 4 lux), placed at 100 cm directly above the olfactometer.

We evaluated the responses of fifth-instar nymphs and adults, so that 80 replicates per treatment were performed. Treatments consisted in 100 mg of faeces, volatiles from a chamber with 150 aggregated bugs, extracts from dynamic aeration of faeces (1 µl from extract; see above) and aggregated insects (1 µl from extract), we also evaluated the extracts (with hexane and methanol) from faeces (1 µl from extract), aggregated insects (1 µl from extract) and bugs' hydrocarbons (1 µl from extract). These volumes were used according to previous experiments performed. In a second set of experiments, 1 µl of two blends of available synthetic standards were evaluated at three different concentrations (approx. 1000, 100 and 10 ng µl^−1^). Blends were performed according to the proportion of compounds found in faeces and aggregated insects volatiles of non-infected bugs in the dynamic aeration technique. In the case of hexanal and heptanal, which were found in SPME experiments, the proportion was the same for octanal. Standards used in the F blend (faeces blend) were: hexanal (10%), heptanal (10%), octanal (10%), 2-ethyl-1-hexanol (20%), nonanal (10%), decanal (10%), nonanoic acid (5%), caprolactam (5%), decanoic acid (10%) and 1-dodecanol (10%). Meanwhile, for the blend AI (aggregated insects) proportions were: hexanal (5%), heptanal (5%), octanal (5%), 2-ethyl-1-hexanol (20%), nonanal (20%), decanal (15%), nonanoic acid (5%), caprolactam (5%), decanoic acid (10%) and 1-dodecanol (10%). Additionally, 1 µl of identified compounds were evaluated individually at 100 ng µl^−1^.

##### 4.2.5.2. Rectangular cage bioassay

We followed the same methodology of Mota et al. [10] with some modifications, to assess thigmotaxis and intense negative phototaxis. A rectangular glass arena (40 × 100 cm) lined with filter paper was used for these tests. We placed on opposite sides of the arena two artificial shelters (corrugated cardboard (20 × 10 cm), folded to generate a 10 cm^2^ shelter with two lateral slits of approx. 0.5 cm in height). In one of the artificial shelters, a piece of filter paper (5 × 5 cm) impregnated with the treatment was introduced, while the other shelter contained a piece of filter paper treated with solvent as control. Firstly, to set the most efficient experimental conditions for the bioassays, we evaluated the response of the insects to conspecific faeces. We performed bioassays at different times of the day with 10 fifth-instar nymphs (most used time for the bioassays is the scotophase), then we evaluated the response of nymphs (third, fourth and fifth instars) and adults. Once we set the experimental conditions, we evaluated the response of a single bug, then we evaluated the response of a group of five insects to different treatments. Bioassays lasted 6 h; fifth-instar nymphs starved for at least 10 days post ecdysis were used. Insects were placed in the centre of the arena 30 min before the beginning of the experiment. Then, they were released; at the end of the experiment, the shelters were carefully removed from the arena and the number of bugs inside each of them was recorded. The illumination of the experimental room was set to a 12 : 12 L/D. In a first trial, treatments were carried out using conspecific faeces (100–150 mg), volatiles from faeces (10 µl from extract; see above), volatiles from aggregated insects (10 µl from extract), hexane and methanol extracts from faeces (25 µl from extract), from cuticle of the bugs (25 µl from extract) and from places where the bugs were aggregated (10 µl from extract). In a second trial, available standards of the identified compounds found in methanol extracts of faeces and aggregated insects and combinations of them were evaluated at 100 ng µl^−1^, using 5 µl. Ten replicates per treatment were performed.

#### 4.2.6. Semi-field bioassay

To test the behavioural responses of bugs to the blend of compounds that we found to possess the aggregation effect, we used a 1 × 1 × 0.50 m wood cage at room conditions (30°C, 90% RH). We placed on opposite sides of the arena two artificial shelters (see above) In one of the artificial shelters, a piece of filter paper (5 × 5 cm) impregnated with the treatment was introduced, while the other shelter contained a piece of filter paper treated with solvent as control. Bioassays lasted 3 h, fifth-instar nymphs and adults of *T. pallidipennis* starved for at least 10 days post ecdysis were used separately. Five insects were placed at the centre of the arena for 30 min, after this time, they were released. At the end of the experiment, the shelters were carefully removed from the arena and the number of bugs inside each of them was recorded. The illumination of the experimental room was set to a 12 : 12 L/D. Treatments were the four-component blend (octanal, nonanal, histidine and histamine) at 1000 ng µl^−1^ against control. Ten replicates per treatment were performed.

#### 4.2.7. Statistical analyses

Data were analysed using the statistical software R version 4.1.3 [38], Levene test for variance homogeneity and Shapiro–Wilk test for normality were applied. Box–Cox transformation [39] was used when needed. Semi-field and rectangular cage bioassays with 10 or 5 bugs were analysed with a *t*-test (two-sided and 95% confidence interval), meanwhile bioassays in the rectangular cage using one insect and ‘Y-tube’ bioassays were analysed with a *G*-test; in all cases non-responder insects were excluded from analysis.

## Data Availability

The data are provided in electronic supplementary material https://doi.org/10.6084/m9.figshare.23631489.v1 [40]. Supplementary material is available online [41].
